# Selective Serotonin Reuptake Inhibitor-Associated Intracranial Hemorrhage: Drug-Specific Risk Patterns and Patient-Level Modifiers

**DOI:** 10.3390/neurolint17070111

**Published:** 2025-07-18

**Authors:** Josef Yayan, Kurt Rasche

**Affiliations:** Department of Internal Medicine, Division of Pulmonary, Allergy and Sleep Medicine, HELIOS Clinic Wuppertal, Witten/Herdecke University, Heusnerstr. 40, 42283 Wuppertal, Germany

**Keywords:** selective serotonin reuptake inhibitors, intracranial hemorrhage, pharmacovigilance, FAERS, anticoagulation, drug safety

## Abstract

Background: Selective serotonin reuptake inhibitors (SSRIs) are among the most commonly prescribed antidepressants and are generally considered safe. However, emerging data suggest a potential association with intracranial hemorrhage (ICH), especially among elderly patients and those on anticoagulation. Methods: We conducted a retrospective pharmacovigilance analysis using data from the U.S. Food and Drug Administration’s Adverse Event Reporting System (FAERS). Reports up to May 2025 listing an SSRI (sertraline, fluoxetine, paroxetine, escitalopram, citalopram, or fluvoxamine) as a suspect or interacting drug and involving an ICH event were included. Disproportionality was assessed using reporting odds ratios (RORs) with 95% confidence intervals. Results: Among 226 eligible ICH cases, sertraline (30.5%), paroxetine (28.8%), and fluoxetine (27.9%) were most frequently implicated. Sertraline showed a strong signal for cerebral hemorrhage (ROR = 4.97), while fluoxetine was associated with subarachnoid hemorrhage (ROR = 4.51). Sertraline had a pronounced signal among patients aged >60 years (ROR = 7.92) and in combination with anticoagulants (ROR = 9.56). Fluoxetine was underrepresented in elderly cases. Given the very small number of fluvoxamine-related cases (n = 2), interpretation should be cautious due to limited statistical power. Gender-stratified analyses showed female predominance in sertraline-related ICH and male predominance for paroxetine. Citalopram demonstrated a potentially protective profile with inverse association with cerebral hemorrhage. Conclusions: This study highlights significant differences in ICH reporting patterns across SSRIs, modified by patient age, gender, and co-medication. These findings underscore the need for individualized SSRI prescribing, particularly in patients receiving anticoagulant therapy particularly in elderly patients and those receiving anticoagulant therapy, where sertraline and fluoxetine may pose increased risk.

## 1. Introduction

Selective serotonin reuptake inhibitors (SSRIs) are among the most widely prescribed classes of antidepressants worldwide and represent the first-line pharmacological treatment for major depressive disorder, generalized anxiety disorder, and several other psychiatric conditions [1]. Their clinical appeal stems from a more favorable safety profile, particularly regarding cardiovascular and anticholinergic side effects, compared to older antidepressants such as tricyclics or MAO inhibitors [2]. As a result, their use has expanded steadily over the past two decades, particularly among elderly populations and individuals with multimorbidity [3].

However, emerging evidence suggests that SSRIs may increase the risk of bleeding, particularly in the gastrointestinal tract [4]. This association is thought to be mediated by the inhibition of serotonin uptake into platelets, thereby impairing platelet aggregation and extending bleeding time [5]. Platelets rely exclusively on serotonin uptake via the serotonin transporter (SERT), as they cannot synthesize it de novo. SSRIs inhibit this uptake, leading to the depletion of intraplatelet serotonin, reduced aggregation, and prolonged bleeding time [5]. While gastrointestinal bleeding associated with SSRIs is well established, data regarding the potential link between SSRIs and intracranial hemorrhage (ICH) remain inconsistent and often rely on limited sample sizes or exploratory signal detection studies, such as the one by Renoux et al. [6].

Several observational studies and meta-analyses have reported a modest but statistically significant association between SSRI use and ICH, particularly in patients concurrently taking antithrombotic agents, as demonstrated in a population-based cohort study by Renoux et al. [6,7] and supported by additional data from Parkin et al. [8]. The combination of SSRIs with anticoagulants or antiplatelet therapy may act synergistically to impair hemostasis and increase the risk of serious bleeding events [9]. Nevertheless, due to the rarity of ICH, absolute risk estimates remain low, and findings across studies have been inconsistent [10].

Another key question pertains to whether all SSRIs carry the same risk or whether differences exist between individual agents. Marked pharmacokinetic and pharmacodynamic differences among SSRIs may lead to divergent safety profiles [11]. These include differences in half-life (e.g., fluoxetine > 72 h vs. paroxetine ~21 h), CYP450 metabolism (e.g., CYP2D6 involvement), and blood–brain barrier penetration, which may impact both efficacy and safety [11]. Furthermore, demographic factors such as patient age and gender, along with clinical parameters such as comorbidity burden and co-medication use, may further modulate bleeding risk. It has been hypothesized that pharmacogenomics and biological sex differences may further modulate these risks, although this was not directly tested in our study.

In this context, pharmacovigilance data from large spontaneous reporting systems, such as the U.S. Food and Drug Administration’s Adverse Event Reporting System (FAERS), offer a valuable source for detecting rare but clinically significant adverse drug reactions, including ICH [12]. Although such systems have inherent limitations, including a lack of exposure denominator data, underreporting, and inconsistent report quality, they allow for the early identification of safety signals that may not be captured in clinical trials or traditional epidemiologic studies [13]. In addition, indication bias may influence the results, particularly in elderly patients who are more likely to receive both SSRIs and antithrombotic agents due to multimorbidity. Disproportionality signals may be affected by factors unrelated to pharmacological risk, such as differences in prescription volumes, stimulated reporting due to litigation or media attention, and regional reporting practices.

The objective of this study was to analyze all reports of ICH associated with SSRI use in the FAERS database, with particular focus on differences among individual SSRIs, patient subgroups (by age and gender), and potential interactions with anticoagulant or antiplatelet therapy. It is important to emphasize that disproportionality analyses based on spontaneous reporting systems like FAERS do not establish causality. These analyses are hypothesis-generating and do not confirm causality. By conducting disproportionality analyses, we aimed to generate real-world evidence that could inform clinical decision-making and guide future research.

## 2. Material and Methods

### 2.1. Data Source

This study was based on data from the U.S. Food and Drug Administration’s Adverse Event Reporting System (FAERS), a publicly accessible pharmacovigilance database designed to monitor adverse drug reactions (ADRs). FAERS includes spontaneous reports submitted by healthcare professionals, patients, and manufacturers. All reports available up to May 2025 were screened. All reports were independently screened by two authors. Discrepancies were resolved through consensus between the reviewers. Preferred terms (PTs) were based on MedDRA v24.1 and included ‘Intracranial hemorrhage’, ‘Subarachnoid hemorrhage’, and ‘Cerebral hemorrhage’, among others. 

### 2.2. Study Design

We conducted a retrospective observational analysis focusing on intracranial hemorrhage (ICH) events associated with selective serotonin reuptake inhibitors (SSRIs). The SSRIs included in this study were sertraline, fluoxetine, paroxetine, escitalopram, citalopram, and fluvoxamine. These six SSRIs were selected based on their widespread clinical use, the availability of sufficient FAERS data, and approval by regulatory agencies for depressive and anxiety disorders.

### 2.3. Inclusion Criteria

Cases were included if they met the following criteria:

- An SSRI was listed as a suspect or interacting drug.
- The adverse event was categorized under MedDRA preferred terms related to ICH (e.g., cerebral hemorrhage, subarachnoid hemorrhage, intracranial hemorrhage unspecified).
- The report contained sufficient information on patient age, sex, or clinical outcomes.

Duplicate entries were excluded using unique case identifiers. Reports with incomplete data were included in overall summaries but excluded from subgroup analyses.

### 2.4. Data Extraction

The following variables were extracted from each eligible report:

- We extracted patient age (both continuous and categorical), sex (male, female, unknown), the implicated SSRI, hemorrhage subtype, event seriousness, and co-medication status.
- Drug information: Implicated SSRI(s).
- Event information: Hemorrhage type (cerebral, subarachnoid, or unspecified), seriousness (death, hospitalization, life-threatening condition, or intervention required).
- Co-medications: Use of anticoagulants, aspirin, or clopidogrel.

All included reports were manually reviewed for relevance and consistency. 

### 2.5. Statistical Analysis

Descriptive statistics were used to summarize the data. Mean and median age, standard deviation (SD), and age ranges were reported for each drug subgroup. Categorical variables were summarized as counts and percentages. To assess relative ICH reporting risk, we conducted disproportionality analyses using reporting odds ratios (RORs) with 95% confidence intervals (CIs). RORs were calculated by comparing the odds of a given event for a specific SSRI with the odds for all other SSRIs combined. An ROR > 1 with a 95% CI not including 1 was considered a signal of disproportionate reporting.

### 2.6. Subgroup Analyses

Disproportionality analyses were also stratified by the following:

- Age group (>60 vs. ≤60 years). The >60 years threshold was selected in accordance with the prior literature on geriatric risk stratification for bleeding events and reflects common clinical definitions of older adults in pharmacovigilance studies.
- Gender (male vs. female).
- Co-medication (with vs. without anticoagulants, aspirin, or clopidogrel).

A two-tailed *p*-value of <0.05 was considered statistically significant. 

### 2.7. Software

Data handling and summary statistics were performed using Microsoft Excel (version 2306, Microsoft Corporation, Redmond, WA, USA). Statistical analyses, including ROR calculations and confidence intervals, were validated using the online tool VassarStats (accessed 4 May 2025, www.vassarstats.net). Data integrity checks were also performed to improve analytical robustness.

## 3. Results

To facilitate interpretation, key findings are summarized in structured tables and visualized in two figures, focusing on drug-specific reporting patterns, subgroup differences, and risk modifiers.

### 3.1. Overview of Reported Cases

A total of 226 cases of intracranial hemorrhage (ICH) associated with the use of selective serotonin reuptake inhibitors (SSRIs) were identified in the FAERS database. The distribution across individual SSRIs was as follows: sertraline (n = 69, 30.5%), paroxetine (n = 65, 28.8%), fluoxetine (n = 63, 27.9%), escitalopram (n = 14, 6.2%), citalopram (n = 13, 5.8%), and fluvoxamine (n = 2, 0.9%). Given the very small number of fluvoxamine-related cases, no meaningful conclusions regarding its safety profile can be drawn.

### 3.2. Demographic Characteristics

As shown in Table 1, sertraline was the most frequently reported SSRI, accounting for 26.1% of all ICH cases. Female patients constituted the majority of reports across most substances, particularly for sertraline (68.1%) and fluvoxamine (100%). The mean age ranged from 49.9 years for paroxetine to 81 years for fluvoxamine, with sertraline cases averaging 72.7 years, reflecting a predominance in older patients (Table 1).

### 3.3. Seriousness of Adverse Events

**Figure 1:**
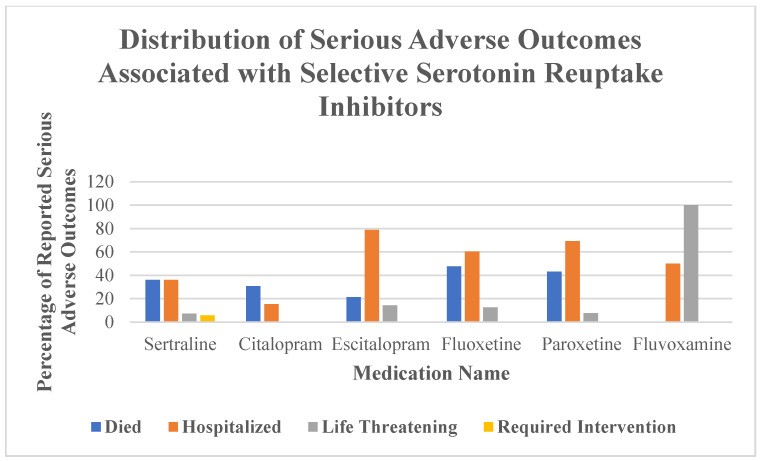
Percentage distribution of serious adverse outcomes reported in association with selective serotonin reuptake inhibitors. Categories include death, hospitalization, life-threatening events, and events requiring medical intervention. Each outcome is shown as a proportion of total serious events for each medication.

The proportion of serious adverse events (death, hospitalization, life-threatening condition, or intervention) was highest for citalopram, escitalopram, paroxetine, and fluvoxamine (each 100%), followed by fluoxetine (98.4%) and sertraline (26.1%) (Table 2). These findings indicate notable differences in clinical severity across agents (Figure 1).

### 3.4. Type of Hemorrhage

**Figure 2:**
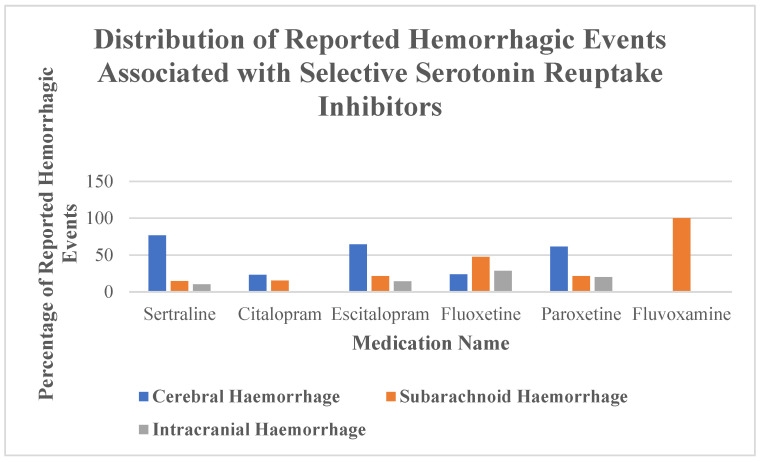
Percentage distribution of reported hemorrhagic events associated with selective serotonin reuptake inhibitors. The chart presents the relative frequency (in %) of reported cases of cerebral hemorrhage, subarachnoid hemorrhage, and intracranial hemorrhage for each individual SSRI. Colors represent different hemorrhage subtypes; error bars are not applicable due to the descriptive nature of the plot.

There was variation in the type of reported ICH. Among sertraline cases, 76.8% involved cerebral hemorrhage. Fluoxetine was disproportionately associated with subarachnoid hemorrhage (47.6%) and intracranial hemorrhage (28.6%). Reports for citalopram and escitalopram included a mix of hemorrhage types but at lower total frequencies (Table 3, Figure 2).

### 3.5. Clinical Outcomes

The highest fatality rates were observed in cases involving fluoxetine (47.6%) and paroxetine (43.1%), followed by sertraline (36.2%). Hospitalization was most common among escitalopram (78.8%) and paroxetine (69.2%) reports. Sertraline cases were more likely to result in fatal outcomes than in hospitalization or intervention (Table 4).

### 3.6. Age Distribution

ICH reports related to sertraline and fluvoxamine were predominantly observed in patients aged ≥60 years (61% and 100%, respectively). In contrast, paroxetine and fluoxetine cases were more evenly distributed across age groups, with 42.9% and 49.2%, respectively, involving individuals under 60 years. As detailed in Table 4, fluoxetine showed a high ROR in older patients (≥65 years) (Table 5).

### 3.7. Co-Medications

Among the 226 cases, 27.5% of sertraline reports involved concurrent anticoagulant use, followed by paroxetine (4.6%) and fluoxetine (3.2%). Aspirin and clopidogrel use were uncommon but mainly reported in sertraline and fluoxetine users. No anticoagulant or antiplatelet co-medication was reported in fluvoxamine cases. As summarized in Table 6, RORs were elevated in patients co-treated with anticoagulants, particularly for citalopram.

### 3.8. Disproportionality Analysis

Disproportionality signals varied across SSRIs and hemorrhage types. Sertraline was significantly associated with cerebral hemorrhage (ROR = 4.97; 95% CI: 2.6–9.5; *p* < 0.0001). Fluoxetine showed significant signals for subarachnoid hemorrhage (ROR = 4.51; *p* < 0.0001) and unspecified intracranial hemorrhage (ROR = 2.56; *p* = 0.0078). Citalopram was inversely associated with cerebral hemorrhage (ROR = 0.24; *p* = 0.0254) (Table 7).

### 3.9. Gender-Stratified Analysis

**Figure 3:**
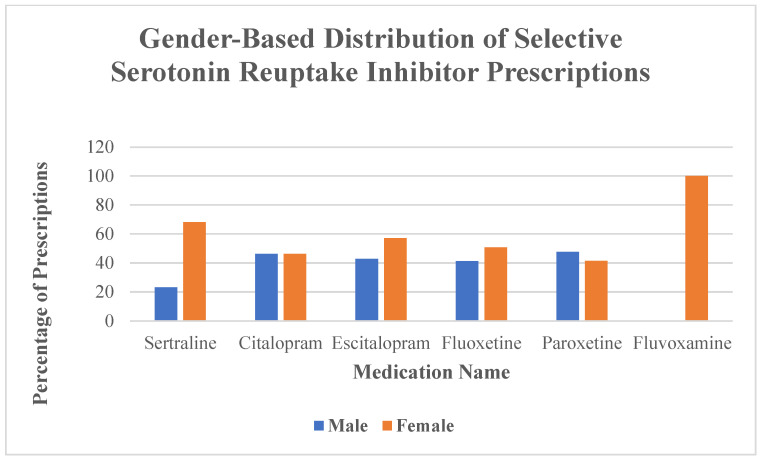
Gender-based distribution of prescriptions for selective serotonin reuptake inhibitors. The chart displays the percentage of prescriptions for each SSRI stratified by gender (blue bars for males, orange bars for females). Medications include sertraline, citalopram, escitalopram, fluoxetine, paroxetine, and fluvoxamine.

Figure 3 shows the gender distribution of prescriptions for individual SSRIs, with sertraline and fluvoxamine being more frequently prescribed to female patients. Sertraline was significantly more often reported in females (ROR = 2.34; *p* = 0.0047), while reporting was significantly lower in males (ROR = 0.39; *p* = 0.003). Although paroxetine showed a numerically higher ROR in males (1.81), this result was not statistically significant (*p* = 0.0468), and therefore no firm conclusions can be drawn (Table 8; Figure 3).

### 3.10. Age-Stratified Analysis

Patients aged >60 years showed a strong signal for sertraline (ROR = 7.92; *p* < 0.0001), whereas fluoxetine was underrepresented in this group (ROR = 0.37; *p* = 0.0036). No significant age-related disproportionality was found for the remaining SSRIs (Table 9).

### 3.11. Interaction with Anticoagulants

Sertraline combined with anticoagulants showed the strongest disproportionality signal (ROR = 9.56; *p* < 0.0001). In contrast, fluoxetine displayed a significantly reduced ROR in the presence of anticoagulation (ROR = 0.20; *p* = 0.0188). No significant signals were observed for aspirin or clopidogrel co-use in any SSRI subgroup (Table 10).

## 4. Discussion

In this pharmacovigilance study using data from the FDA Adverse Event Reporting System (FAERS) database, we identified a total of 226 reports of intracranial hemorrhage (ICH) associated with the use of selective serotonin reuptake inhibitors (SSRIs). The results reveal considerable differences between individual SSRIs in terms of reported frequency, patient demographics, hemorrhage subtypes, and clinical outcomes.

Sertraline, paroxetine, and fluoxetine were the most frequently implicated agents. Table 1 shows that ICH cases associated with sertraline were predominantly reported in older individuals and female patients, a distribution that may reflect prescribing trends or differential vulnerability. While their dominance likely reflects broader prescription patterns [12], our disproportionality analysis suggests that these agents may also differ in their intrinsic bleeding risk. However, these findings may in part reflect prescription patterns. For instance, if sertraline is more commonly prescribed to elderly patients or those requiring anticoagulation, the observed disproportionality signals may be confounded by differential exposure rather than intrinsic pharmacological risk. Specifically, sertraline showed a strong signal for cerebral hemorrhage, whereas fluoxetine was disproportionately associated with subarachnoid and unspecified intracranial hemorrhage. In contrast, citalopram appeared to be inversely associated with cerebral hemorrhage, although the total number of cases was small. Due to the extremely limited number of reports involving fluvoxamine, no reliable inferences can be made about its association with ICH.

This is consistent with prior observational and pharmacovigilance studies reporting elevated ICH risk in elderly or anticoagulated SSRI users [13,14]. It has been hypothesized that the biological mechanism involves reduced serotonin uptake into platelets, leading to impaired aggregation and increased bleeding tendency, although this was not directly assessed in our study [15]. Co-medication with anticoagulants or antiplatelet agents may amplify this risk [16], as supported by our finding that sertraline combined with anticoagulants yielded a markedly elevated ROR of 9.56.

Older patients appear to be particularly susceptible to SSRI-associated ICH. Our age-stratified disproportionality analysis revealed a highly significant signal for sertraline in patients over 60 years of age (ROR = 7.92), consistent with prior evidence that age is an independent risk factor for spontaneous ICH [17]. The lower representation of fluoxetine among elderly patients may reflect prescribing behavior or underlying pharmacokinetic considerations, such as its long half-life [18]. In addition to its prolonged half-life, fluoxetine’s high permeability across the blood–brain barrier may increase its interaction with neurovascular structures, potentially contributing to the observed association with subarachnoid hemorrhage. Moreover, serotonin plays a role in modulating endothelial function and vascular tone. By interfering with serotonergic signaling in the cerebral vasculature, SSRIs may impair cerebrovascular autoregulation, further increasing susceptibility to hemorrhagic events, particularly in vulnerable populations [18].

The gender-stratified analyses revealed that women were overrepresented in sertraline-related ICH reports (ROR = 2.34), whereas paroxetine was disproportionately reported in men (ROR = 1.81). These findings may relate to gender-specific prescription patterns, pharmacokinetics, and biological differences in platelet reactivity or vascular structure [19,20]. However, FAERS data do not allow for direct adjustment for exposure prevalence by sex, and these observations must be interpreted with caution.

Spontaneous reporting systems like FAERS have well-known limitations, including underreporting, differential reporting bias, and a lack of denominator data [21,22]. Nonetheless, such systems are useful for generating hypotheses and detecting rare but serious adverse events that may not be captured in randomized controlled trials. Notably, our study provides novel insights by systematically differentiating between types of ICH, exploring demographic modifiers, and highlighting potential drug–drug interactions.

Our results support prior studies demonstrating an increased bleeding risk when SSRIs are used in combination with antithrombotic agents [23]. Given the widespread use of SSRIs in patients with cardiovascular or cerebrovascular conditions—many of whom are already on anticoagulation—these findings have clear clinical relevance. For example, patients with atrial fibrillation, mechanical heart valves, or prior thromboembolic events often require anticoagulation, and the addition of SSRIs may further increase bleeding risk [24].

Although the absolute incidence of ICH in SSRI users remains low, the consequences of such events are often devastating. Careful risk–benefit assessment is warranted when prescribing SSRIs to high-risk individuals, especially elderly patients and those on oral anticoagulants or antiplatelet therapy. In such cases, alternative antidepressants with a more favorable bleeding profile should be considered, or at minimum, close monitoring should be implemented.

Further research is needed to confirm these associations and quantify absolute risk. Large-scale population-based studies using linked prescription, hospital, and mortality databases could help identify causality and clarify the temporal sequence between drug exposure and hemorrhage onset [25]. Advanced data mining methods, including machine learning and Bayesian signal detection, may also enhance pharmacovigilance efforts [26].

Despite its limitations, FAERS remains a valuable tool for early signal detection and pharmacovigilance surveillance. The heterogeneity in bleeding risk observed across individual SSRIs in this study challenges the notion that the SSRI class is pharmacologically homogeneous in terms of safety [27]. Instead, our results support the emerging view that safety profiles must be assessed at the substance level rather than the drug class level [28].

In conclusion, sertraline and fluoxetine were associated with elevated reporting signals for ICH in FAERS, particularly in elderly patients and those on anticoagulants. These findings underscore the importance of personalized prescribing, post-marketing surveillance, and further investigation into the comparative safety of antidepressants. As the use of SSRIs continues to rise globally, especially among high-risk populations, these safety concerns should not be overlooked [29,30].

Finally, our findings also highlight the need for regulatory bodies to consider drug-specific warnings rather than class-wide alerts when new safety data emerge. While SSRIs are widely considered a homogeneous pharmacological class, differences in molecular structure, metabolism, and off-target effects may result in divergent safety profiles. Post-marketing surveillance programs should therefore aim to stratify risk at the compound level. Furthermore, improved clinician education on pharmacovigilance principles could help bridge the gap between early signal detection and clinical practice. The integration of electronic health records with real-time pharmacovigilance alerts might enable more timely interventions and individualized patient monitoring, especially in high-risk groups. These findings raise important considerations for practical prescribing decisions in clinical care. Ongoing pharmacovigilance efforts and interprofessional collaboration remain vital in optimizing patient safety.

While our study focused exclusively on SSRIs, it would be valuable to compare the observed bleeding signals to those reported with other antidepressant classes, such as serotonin–norepinephrine reuptake inhibitors (SNRIs) and tricyclic antidepressants (TCAs). Prior research has suggested that TCAs may carry a lower bleeding risk, whereas some SNRIs, such as venlafaxine, may also inhibit platelet function. Including these agents as comparators in future studies could provide a more comprehensive safety profile for clinicians.

### 4.1. Clinical Implications

The findings of this study have direct clinical relevance for physicians prescribing SSRIs, particularly in vulnerable populations. Given the observed association between sertraline and cerebral hemorrhage, and between fluoxetine and subarachnoid or unspecified intracranial bleeding, prescribers should consider individual bleeding risk when selecting an antidepressant. This is especially pertinent in elderly patients and those receiving concomitant anticoagulation or antiplatelet therapy, such as warfarin, direct oral anticoagulants (DOACs), aspirin, or clopidogrel.

For patients with multiple risk factors for bleeding—such as advanced age, hypertension, a history of stroke, or dual antithrombotic therapy—clinicians may consider the following:

- Choosing SSRIs with lower bleeding signals (e.g., citalopram, based on this study);
- Close monitoring during the initiation phase, especially within the first weeks when risk may be highest;
- The regular review of anticoagulation indication and dosing;
- Educating patients and caregivers for them to promptly recognize warning signs of neurological deterioration.

In psychiatric patients with cardiovascular comorbidity, multidisciplinary collaboration between psychiatrists, internists, and neurologists may help tailor safer and more effective treatment strategies. Pharmacogenetic testing and individualized risk modeling could also improve safety profiles in the future.

These implications highlight the importance of moving beyond a one-size-fits-all approach in antidepressant prescribing and emphasize the need for vigilance in pharmacotherapy of high-risk groups.

Beyond prescribing choices, these results emphasize the importance of shared decision-making. Physicians should engage patients in discussions about the potential bleeding risks of SSRIs, particularly when alternative treatment options exist. Patient-reported symptoms such as sudden headache, confusion, visual disturbance, or focal neurological signs should prompt immediate medical evaluation. Establishing clear follow-up plans, especially during the early treatment phase or during dose changes, can improve the early detection of adverse events. Furthermore, deprescribing strategies should be considered when the indication for SSRI use is no longer present or when bleeding risk outweighs potential benefit.

### 4.2. Limitations

This study has several important limitations inherent to the use of spontaneous reporting systems such as FAERS. First, underreporting is a well-documented phenomenon in pharmacovigilance databases, and the true number of intracranial hemorrhage (ICH) cases related to SSRI use may be substantially higher than captured. Conversely, reporting bias—driven by media attention, recent publications, or litigation—can lead to the overrepresentation of certain drugs or events, distorting relative signal strengths. Moreover, FAERS does not provide consistent information on the time interval between drug initiation and adverse events, limiting our ability to evaluate temporal associations. Furthermore, information on SSRI dosage and duration of treatment was not available. These factors are known to influence bleeding risk and may have contributed to the observed heterogeneity in adverse event reporting.

Second, the lack of denominator data (i.e., total number of patients exposed to each SSRI) precludes the calculation of incidence rates or absolute risk estimates. As a result, disproportionality analysis can only detect relative reporting signals, which are hypothesis-generating rather than confirmatory in nature.

Third, clinical details are frequently missing or incomplete in FAERS reports. Data on drug dosage, the duration of exposure, the temporal relationship between drug initiation and ICH onset, imaging confirmation, underlying comorbidities, and laboratory parameters are typically not available. This lack of granularity limits causal interpretation and makes it difficult to distinguish true adverse drug reactions from confounding by indication or comorbidity.

Fourth, co-medications may not be consistently or accurately reported, leading to the possible underestimation of interaction effects, particularly for anticoagulants, antiplatelet agents, or nonsteroidal anti-inflammatory drugs (NSAIDs), which are known to elevate bleeding risk.

Finally, spontaneous reporting systems are not designed for comparative effectiveness or safety analyses between drugs. Differences in reporting rates between individual SSRIs may reflect variations in market share, prescriber preference, patient populations, or regional reporting practices, rather than true pharmacological differences.

Despite these limitations, FAERS remains a valuable tool for detecting rare but serious adverse events and generating safety signals that can inform future research and regulatory decision-making.

## 5. Conclusions

This pharmacovigilance study identified notable safety signals for intracranial hemorrhage (ICH) associated with selective serotonin reuptake inhibitors (SSRIs), particularly sertraline and fluoxetine. Disproportionality analyses suggest that individual SSRIs may differ in their hemorrhagic risk profiles, with sertraline being significantly associated with cerebral hemorrhage and fluoxetine with subarachnoid and unspecified intracranial bleeding. These risks appear to be modified by patient age, sex, and the concomitant use of anticoagulants.

The markedly increased reporting odds for ICH in elderly patients and those co-treated with anticoagulants highlight clinically relevant safety concerns that warrant careful consideration during SSRI prescribing. Although these findings do not establish causality, they underscore the importance of personalized risk assessment, particularly in vulnerable populations.

Further studies using population-based data and controlled designs are needed to confirm these associations, quantify absolute risks, and guide clinical decision-making. Until then, clinicians should remain vigilant when prescribing SSRIs, especially in patients with elevated bleeding risk, and consider alternative treatment strategies when appropriate.

Until such data become available, the close monitoring of neurological warning signs, cautious risk stratification, and judicious prescribing are key. Clinicians must remain alert to signs of ICH, especially in patients presenting with atypical neurological symptoms during SSRI therapy. Enhanced pharmacovigilance remains essential to ensure the safe use of SSRIs globally. Early neuroimaging and interdisciplinary evaluation should be considered in high-risk patients reporting such symptoms, particularly during the initial phase of SSRI treatment or in combination with anticoagulants.

## Figures and Tables

**Table 1:** Demographic characteristics of intracranial hemorrhage (ICH) cases by SSRI. Variables include the drug substance (active ingredient), the total number of reports, gender distribution (absolute and relative frequencies), and age-related information. The mean age is reported with standard deviation (SD), and the median age is shown along with the corresponding minimum and maximum values.

Drug Substance	Number	Male (%)	Female (%)	Unspecified Gender (%)	Mean Age ± Standard Deviation (SD)	Median Age (Minimum–Maximum Age)
Sertraline	69	16 (23.2)	47 (68.1)	6 (8.7)	72.7 ± 16.9	79 (13–91)
Citalopram	13	6 (46.2)	6 (46.2)	1 (7.7)	51.4 ± 23.7	55 (1–81)
Escitalopram	14	6 (42.9)	8 (57.1)	0	56.6 ± 24.8	51.9 (1–91)
Fluoxetine	63	26 (41.3)	32 (50.8)	5 (7.9)	53.9 ± 18.4	54 (14–85)
Paroxetine	65	31 (47.7)	27 (41.5)	7 (10.8)	49.9 ± 27.8	55 (1–90)
Fluvoxamine	2	0	2 (100)	0	81 ± 0	81(81–81)

**Table 2:** Severity of reported intracranial hemorrhage cases associated with each SSRI. The total number of reports is presented alongside the number and percentage of events classified as serious. In the FAERS database, serious events typically include outcomes such as death, hospitalization, life-threatening conditions, or other medically significant events.

Drug Substance	Number	Seriousness of Event (%)
Sertraline	69	18 (26.1)
Citalopram	13	13 (100)
Escitalopram	14	14 (100)
Fluoxetine	63	62 (98.4)
Paroxetine	65	65 (100)
Fluvoxamine	2	2 (100)

Note: Seriousness percentages may be influenced by reporting completeness. Lower percentages (e.g., for sertraline) may reflect underdocumentation of severity rather than true differences.

**Table 3:** Distribution of reported bleeding types associated with each SSRI in cases of intracranial hemorrhage. The classification is based on MedDRA terminology and includes the number of reports for cerebral hemorrhage, subarachnoid hemorrhage, and unspecified intracranial hemorrhage. It should be noted that multiple bleeding types may be reported for a single case.

Drug Substance	Number	Cerebral Hemorrhage (%)	Subarachnoid Hemorrhage (%)	Intracranial Hemorrhage (%)
Sertraline	69	53 (76.8)	10 (14.5)	7 (10.1)
Citalopram	13	3 (23.1)	2 (15.4)	0
Escitalopram	14	9 (64.3)	3 (21.4)	2 (14.3)
Fluoxetine	63	15 (23.8)	30 (47.6)	18 (28.6)
Paroxetine	65	40 (61.5)	14 (21.5)	13 (20.0)
Fluvoxamine	2	0	2 (100)	0

**Table 4:** Overview of clinical outcomes reported in association with intracranial hemorrhage during SSRI treatment. The outcomes include the number of cases resulting in death, hospitalization, or life-threatening conditions and those requiring medical or surgical intervention. The values reflect the severity and potential clinical impact of reported adverse events, as documented in the FAERS database.

Drug Substance	Number	Died	Hospitalized	Life-Threatening	Required Intervention
Sertraline	69	25 (36.2)	25 (36.2)	5 (7.2)	4 (5.8)
Citalopram	13	4 (30.8)	2 (15.4)	0	0
Escitalopram	14	3 (21.4)	11 (78.8)	2 (14.3)	0
Fluoxetine	63	30 (47.6)	38 (60.3)	8 (12.7)	0
Paroxetine	65	28 (43.1)	45 (69.2)	5 (7.7)	0
Fluvoxamine	2	0	1 (50.0)	2 (100)	0

**Table 5:** Distribution of patient age groups in reports of intracranial hemorrhage during SSRI treatment. The number of cases is categorized into standard age ranges (0–20, 20–40, 40–60, 60–80, and 80–100), with an additional column for cases in which the patient’s age was not specified. These data provide insights into age-related reporting patterns and potential age-dependent risks.

Drug Substance	Number	0–20 Years	20–40 Years	40–60 Years	60–80 Years	80–100 Years	Not Specified
Sertraline	69	1 (1.4)	2 (2.9)	4 (5.8)	19 (27.5)	23 (33.3)	20 (29.0)
Citalopram	13	1 (7.7)	1 (7.7)	3 (23.1)	4 (30.8)	1 (7.7)	3 (23.1)
Escitalopram	14	1 (7.1)	4 (28.6)	1 (7.1)	4 (28.6)	1 (7.1)	3 (21.4)
Fluoxetine	63	1 (1.6)	10 (15.9)	20 (31.7)	15 (23.8)	4 (6.3)	13 (20.6)
Paroxetine	65	11 (16.9)	3 (4.6)	14 (21.5)	18 (27.7)	4 (6.2)	15 (23.1)
Fluvoxamine	2	0	0	0	0	2 (100)	0

**Table 6:** An overview of concomitant antithrombotic therapy in cases of SSRI-associated intracranial hemorrhage is provided below. The table includes the number of patients who were also receiving anticoagulation therapy, aspirin, or clopidogrel (Plavix). These co-medications may influence bleeding risk and are relevant for interpreting potential drug interactions.

Drug Substance	Number	Anticoagulation	Aspirin	Clopidogrel
Sertraline	69	19 (27.5)	5 (7.2)	1 (1.4)
Citalopram	13	1 (7.7)	1 (7.7)	0
Escitalopram	14	0	0	0
Fluoxetine	63	2 (3.2)	4 (6.3)	1 (1.6)
Paroxetine	65	3 (4.6)	2 (3.1)	0
Fluvoxamine	2	0	0	0

**Table 7:** Disproportionality analysis of selected SSRIs for three types of intracranial bleeding reported in FAERS, including reporting odds ratios (RORs), 95% confidence intervals (CIs), and *p*-values compared to all other SSRIs. An ROR > 1 suggests a higher reporting rate. Fluoxetine was significantly associated with subarachnoid and intracranial hemorrhage, while sertraline showed a strong signal for cerebral hemorrhage but a lower reporting rate for subarachnoid events. Citalopram was in versely associated with cerebral hemorrhage. Results for fluvoxamine and some endpoints could not be calculated due to small case numbers.

Drug Substance	Cerebral Hemorrhage, ROR (95% CI), *p*-Value	Subarachnoid Hemorrhage, ROR (95% CI), *p*-Value	Intracranial Hemorrhage, ROR (95% CI), *p*-Value
Sertraline	4.97 (2.6–9.5), **<0.0001**	0.35 (0.17–0.74), 0.0050	0.42 (0.18–1.01), **0.0485**
Citalopram	0.24 (0.07–0.92), **0.0254**	0.47 (0.10–2.21), 0.3714	n/a
Escitalopram	1.0 (0.37–2.7), 1.0	0.72 (0.20–2.69), 0.7638	0.76 (0.16–3.55), 0.7297
Fluoxetine	0.17 (0.09–0.32), **<0.0001**	4.51 (2.41–8.46), **<0.0001**	2.56 (1.26–5.20), **0.0078**
Paroxetine	1.58 (0.88–2.85), 0.1261	0.67 (0.34–1.32), 0.2401	1.24 (0.59–2.59), 0.5647
Fluvoxamine	0 (n/a–n/a), 0.2189	0 (n/a–n/a), 0.0700	n/a

n/a = not available; value not calculable due to insufficient data (n < 5). Note: Significant *p*-values are highlighted in bold.

**Table 8:** Sex-specific disproportionality analyses for selective serotonin reuptake inhibitors (SSRIs) based on FAERS data. The table shows the reporting odds ratios (RORs) with 95% confidence intervals (CIs) and *p*-values for male and female patients separately. Each SSRI was compared to all other SSRIs as a reference group. An ROR > 1 indicates a higher-than-expected proportion of reports from that sex; an ROR < 1 indicates a lower-than-expected reporting frequency. Sertraline was significantly more often reported in female patients (ROR = 2.34; *p* = 0.0047) and reported significantly less in males (ROR = 0.39; *p* = 0.003), suggesting a sex-related reporting pattern. In contrast, paroxetine showed a significantly increased male-specific ROR (1.81; *p* = 0.0468) and a reduced female-specific ROR (0.49; *p* = 0.0171). These findings may reflect differences in usage, susceptibility, or reporting behavior by gender. Other SSRIs showed no statistically significant sex-related disproportionality. Fluvoxamine could not be evaluated due to insufficient data.

Drug Substance	ROR (Male)	95% CI (Male)	*p*-Value (Male)	ROR (Female)	95% CI (Female)	*p*-Value (Female)
Sertraline	0.39	0.2–0.73	**0.003**	2.34	1.29–4.24	**0.0047**
Citalopram	1.45	0.47–4.48	0.5125	0.72	0.23–2.2	0.5597
Escitalopram	1.26	0.42–3.77	0.6756	1.15	0.38–3.42	0.8065
Fluoxetine	1.24	0.68–2.24	0.4802	0.84	0.47–1.5	0.5499
Paroxetine	1.81	1.01–3.25	**0.0468**	0.49	0.28–0.89	**0.0171**
Fluvoxamine	n/a	n/a–n/a	n/a	n/a	n/a–n/a	n/a

n/a = not available; value not calculable due to insufficient data (n < 5). Note: Significant *p*-values are highlighted in bold.

**Table 9:** Disproportionality analysis of SSRI-related intracranial hemorrhage reports in patients aged >60 versus ≤60 years, based on FAERS data. The reporting odds ratios (RORs), 95% confidence intervals (CIs), and *p*-values were calculated for each drug using all other SSRIs as the reference group. An ROR > 1 suggests a disproportionate number of reports in patients >60 years. Sertraline shows a strong and statistically significant overrepresentation of reports in older patients (ROR = 7.92; *p* < 0.0001). Conversely, fluoxetine shows a significantly lower proportion of such reports (ROR = 0.37; *p* = 0.0036). No significant disproportionality was observed for the other SSRIs. Analysis for fluvoxamine was not possible due to insufficient case numbers.

Drug Substance	ROR (>60 y)	95% CI	*p*-Value
Sertraline	7.92	3.3–19.03	**<0.0001**
Citalopram	0.8	0.22–2.87	0.7317
Escitalopram	0.66	0.19–2.24	0.5003
Fluoxetine	0.37	0.19–0.73	0.0036
Paroxetine	0.53	0.27–1.03	0.0579
Fluvoxamine	n/a	n/a	n/a

n/a = not available; value not calculable due to insufficient data (n < 5). Note: Significant *p*-values are highlighted in bold.

**Table 10:** Subgroup disproportionality analysis of co-medications (anticoagulation, aspirin, clopidogrel) in SSRI-associated intracranial hemorrhage. Reporting odds ratios (RORs) with 95% confidence intervals and *p*-values compare the frequency of each co-medication to all other SSRIs. The table presents reporting odds ratios (RORs) with 95% confidence intervals (CIs) and *p*-values for each co-medication type (anticoagulation, aspirin, and clopidogrel), comparing each SSRI to all other SSRIs as the reference group. An ROR > 1 indicates an overrepresentation of the respective co-medication. Statistically significant values (*p* < 0.05) are considered potential safety signals. Among the evaluated SSRIs, sertraline showed a statistically significant overrepresentation of reports involving concomitant anticoagulation therapy (ROR = 9.56; *p* < 0.0001), suggesting a potential interaction or heightened bleeding risk. In contrast, fluoxetine was associated with a significantly lower proportion of anticoagulated cases (ROR = 0.20; *p* = 0.0188). No significant disproportionality was observed for aspirin or clopidogrel co-medication in any SSRI group. Several SSRIs could not be assessed for certain co-medications due to absence of reported cases.

Drug Substance	Co-medication	ROR	95% CI	*p*-Value
Sertraline	Anticoagulation	9.56	3.62–25.28	**<0.0001**
Citalopram	Anticoagulation	0.66	0.08–5.27	0.6899
Escitalopram	Anticoagulation	n/a	n/a	n/a
Fluoxetine	Anticoagulation	0.2	0.05–0.87	**0.0188**
Paroxetine	Anticoagulation	0.31	0.09–1.06	**0.0496**
Fluvoxamine	Anticoagulation	n/a	n/a	n/a
Sertraline	Aspirin	1.67	0.51–5.47	0.3894
Citalopram	Aspirin	1.53	0.18–12.86	0.6931
Escitalopram	Aspirin	n/a	n/a	n/a
Fluoxetine	Aspirin	1.31	0.38–4.53	0.6648
Paroxetine	Aspirin	0.48	0.1–2.25	0.3415
Fluvoxamine	Aspirin	n/a	n/a	n/a
Sertraline	Clopidogrel	2.29	0.14–37.22	0.5482
Citalopram	Clopidogrel	n/a	n/a	n/a
Escitalopram	Clopidogrel	n/a	n/a	n/a
Fluoxetine	Clopidogrel	2.61	0.16–42.42	0.4834
Paroxetine	Clopidogrel	n/a	n/a	n/a
Fluvoxamine	Clopidogrel	n/a	n/a	n/a

n/a = not available; value not calculable due to insufficient data (n < 5). Note: Significant *p*-values are highlighted in bold.

## Data Availability

The data used in this study are derived from a publicly accessible pharmacovigilance database and are available from the corresponding author upon reasonable request.
